# Editorial: Neuroscience and neurological machine learning for cognitive assessment: advancements, challenges, and future directions

**DOI:** 10.3389/fnagi.2024.1406184

**Published:** 2024-05-29

**Authors:** Gautam Srivastava, Dan Wu, Chao Liu, Jinping Xu

**Affiliations:** ^1^Brandon University, Brandon, MB, Canada; ^2^Shenzhen Institute of Advanced Technology, Chinese Academy of Sciences (CAS), Shenzhen, China; ^3^University of Maryland, Baltimore, MD, United States

**Keywords:** cognitive, neuroscience, machine learning (ML), neurological, assessment

Neuroscience provides valuable insights into the neural mechanisms underlying cognitive aging and how cognitive learning can promote healthy aging. Cognitive assessment plays a critical role in aging neuroscience because it helps researchers understand how aging affects cognitive function. As individuals age, they may experience cognitive decline, which can lead to difficulties with memory, attention, and decision-making. Cognitive evaluation also allows researchers to measure these changes in cognitive function over time and to identify factors that may contribute to cognitive decline. However, traditional cognitive examination tools have limitations, including limited sensitivity to subtle changes in cognitive functioning and the inability to account for individual differences in cognitive ability. The emerging field of neurological learning offers promise for addressing these limitations, by incorporating machine learning techniques into neurocognitive testing and developing automated and sustainable solutions that can quickly and accurately assess an individual's cognitive health.

Neurological machine learning techniques show promise for improving cognitive assessment, but there are challenges in detecting subtle changes in cognitive function over time and traditional methods rely on subjective interpretation. These models provide objective and accurate assessments by using data-driven approaches to identify cognitive impairment, leading to personalized and accurate assessments, better treatment outcomes, and improved patient care. Further research is needed to address limitations and explore the full potential of neurological learning models in cognitive assessment.

Neurolearning, on the other hand, is a relatively new field that focuses on the use of neuroscientific techniques and tools to improve learning and cognitive performance. Together, these two fields have the potential to revolutionize the way we understand and enhance human learning. This Research Topic aims to explore the intersection of cognitive learning and neurolearning, highlighting emerging trends and applications in the field. In light of these facts and developments, this Research Topic collected original research and review articles with a focus on investigating and sharing groundbreaking ideas, approaches, hypotheses, and practices centered on cognitive healthcare applications. The collection received a total of 20 submissions, of which seven manuscripts were chosen for the final collection. The following few paragraphs summarize their contributions.

In “*Towards human-like walking with biomechanical and neuro-control features: personalized attachment point optimization method of cable-driven exoskeleton*,” Chen et al. leverage principles from neuroscience and biomechanical analysis to select attachment points for cable-driven soft exoskeletons. By extracting key features of human movement, the objective is to develop a subject-specific design methodology that provides precise and personalized support in optimizing the attachment points of the cable-driven exoskeleton to achieve natural gait, energy efficiency, and controllable muscle coordination in the domain of human mobility and rehabilitation. To achieve this, the study first analyzes experimental human gait data and extracts biomechanical features. These features are then used to generate trajectories that allow for better natural movement under complete control of the cable-driven exoskeleton.

In “*A screening method for mild cognitive impairment in elderly individuals combining bioimpedance and MMSE*,” Jun et al. investigate a screening method for mild cognitive impairment (MCI) that combines bioimpedance features and the Korean Mini-Mental State Examination (K-MMSE) score. Data were collected from 539 subjects aged 60 years or older at the Gwangju Alzheimer's & Related Dementias (GARD) Cohort Research Center; a total of 470 participants were used for the analysis, including 318 normal controls and 152 MCI participants. The authors focused on measuring bioimpedance, the K-MMSE, and the Seoul Neuropsychological Screening Battery (SNSB-II). Finally, they developed a multiple linear regression model to predict MCI by combining bioimpedance variables and the K-MMSE total score and compared the accuracy of the model with SNSB-II domain scores by the area under the receiver operating characteristic curve (AUROC).

In “*A deep learning model for brain age prediction using minimally preprocessed T1w-images as input*,” Dartora et al. develop and validate a convolutional neural network (CNN)-based biological brain age prediction model that uses a single T1w MRI preprocessing step when applying the model to external datasets, to simplify implementation and increase accessibility in research settings. Their model only requires rigid image registration to the MNI space, which is an advantage compared to previous methods that require more preprocessing steps, such as feature extraction.

In “*Human-to-monkey transfer learning identifies the frontal white matter as key determinant for predicting monkey brain age*,” He et al. focused on the application of artificial intelligence (AI) to summarize a whole-brain magnetic resonance image (MRI) into an effective “brain age” metric that can provide a holistic, individualized, and objective view of how the brain interacts with various factors (e.g., genetics, lifestyle) during aging. Brain age prediction using deep learning (DL) has been widely used to quantify the developmental status of the human brain, but its wider application for biomedical purposes is under criticism for requiring large sample sizes and complicated interpretability.

In “*The use of the integrated cognitive assessment (ICA) to improve the efficiency of primary care referrals to memory services in the accelerating dementia pathway technologies (ADePT) study*,” Modarres et al. summarize the Accelerating Dementias Pathways Technologies (ADePT) study, which was initiated to address this challenge and develop a real-world evidence basis to support the adoption of ICA as an inexpensive screening tool for the detection of cognitive impairment and improving the efficiency of the dementia care pathway. The primary outcome of the study compared GP referral with specialist diagnosis of mild cognitive impairment (MCI) and dementia. Of those the GP referred to memory clinics, 78% were necessary referrals and ~22% were unnecessary referrals or patients who should have been referred to other services as they had disorders other than MCI/dementia.

In “*Machine learning-based prediction of post-stroke cognitive status using electroencephalography-derived brain network attributes*,” Lee et al. enrolled consecutive stroke patients who underwent both EEG during the acute stroke phase and cognitive assessments 3 months post-stroke. The authors pre-processed the acute stroke EEG data to eliminate low-quality epochs, then performed independent component analysis and quantified network characteristics using iSyncBrain^®^. Cognitive function was evaluated using the Montreal Cognitive Assessment (MoCA). They also initially categorized participants based on the lateralization of their lesions and then developed machine learning models to predict cognitive status in the left and right hemisphere lesion groups.

In “*Altered brain functional connectivity in vegetative state and minimally conscious state*,” Yang et al. used resting-state functional magnetic resonance imaging data to evaluate the weighted brain functional networks for normal control subjects and patients with a vegetative state (VS) and a minimally conscious state (MCS). The global and local network characteristics of each group were analyzed. A back-propagation neural network was employed to identify MCS and VS patients.

The editors would like to express their deepest appreciation to all the authors and reviewers for their timely contributions that made this Research Topic a great success. The editors have done their best to add potential value to the existing literature in this domain. They are very grateful to the Editor-in-Chief of Frontiers in Aging Neuroscience and the Journal staff for their support and for offering us the privilege of editing a Research Topic in this prestigious journal. The editors hope that the novel and scientific contributions presented in this Research Topic will add value to the international scientific research community.

## Author contributions

GS: Writing – original draft, Writing – review & editing. DW: Writing – review & editing. CL: Writing – review & editing. JX: Writing – review & editing.

